# Growth performance, nutrient utilization, and feed efficiency in broilers fed *Tithonia diversifolia* leaf meal as substitute of conventional feed ingredients in Mizoram

**DOI:** 10.14202/vetworld.2016.444-449

**Published:** 2016-05-06

**Authors:** Rajat Buragohain

**Affiliations:** Department of Animal Nutrition, College of Veterinary Sciences and Animal Husbandry, Central Agricultural University, Selesih, Aizawl, Mizoram, India

**Keywords:** broiler, growth, Mizoram, nutrient digestibility, *Tithonia diversifolia* leaf meal

## Abstract

**Aim:**

The study was for assessment of growth performance, nutrient utilization, and feed efficiency in broilers fed rations with varying levels of *Tithonia diversifolia* leaf meal (TDLM) as a substitute of conventional feed ingredients in Mizoram.

**Materials and Methods:**

A total of 180, 1-day-old broiler chicks were randomly divided into six homogeneous groups and fed rations incorporated with TDLM (TDLM at 0% [TDLM-0], 2% [TDLM-2], 4% [TDLM-4], 6% [TDLM-6], 8% [TDLM-8], and 10% [TDLM-10] level as substitute of conventional feed ingredients) for 6 weeks. The chicks were reared in battery brooders for the first 2 weeks, and thereafter, in well-ventilated deep litter house following standard management protocols. Feed and drinking water were provided *ad libitum* to all the groups throughout the experiment. The daily feed intake and weekly body weight gain were recorded, and a metabolic trial for 3 days was conducted at the end of the 6^th^ week.

**Results:**

Feed consumption decreased for inclusion of TDLM but without any significant differences, except during the 3^rd^ week where it reduced significantly (p<0.05) at and above 6% TDLM in the ration. The average body weight gain decreased significantly (p<0.05) above 6% TDLM inclusion. The average body weights at 7^th^, 14^th^, and 21^st^ day of age reduced significantly (p<0.05) from 4% to 10% TDLM inclusion level but was statistically non-significant up to 4% TDLM at 28^th^, 35^th^, and 42^nd^ day of age. Body weight at 42^nd^ day of age was 1624.72±30.52, 1616.66±17.84, 1592.60±19.24, 1404.61±17.76, 1188.29±17.67, and 1054.33±18.81 gin TDLM-0, TDLM-2, TDLM-4, TDLM-6, TDLM-8, and TDLM-10, respectively. The digestibility of nutrients decreased with increased inclusion level of TDLM. The digestibility coefficient of dry matter, crude protein, ether extract, and nitrogen free extract were significantly higher in TDLM-0, but crude fiber digestibility was comparable without any significant difference among the groups. Feed conversion ratio (FCR) at 42^nd^ day of age was 2.17±0.15, 2.17±0.15, 2.13±0.13, 2.46±0.16, 2.66±0.11, and 3.96±0.10 for TDLM-0, TDLM-2, TDLM-4, TDLM-6, TDLM-8, and TDLM-10, respectively, was statistically non-significant up to 4% TDLM inclusion level.

**Conclusion:**

Considering the insignificant effects on growth rate, FCR, and body weight at 42^nd^ day of age, it was concluded that TDLM could be incorporated up to 4% level as substitute of the conventional feed ingredients for broilers reared under deep litter system of management in Mizoram.

## Introduction

Broiler farming is a popular economic activity throughout Mizoram for the tremendous demand of its meat in the state. The broiler is sold at Rs. 170-200/kg live weight and Rs. 250-280/kg dressed weight (Market price during 2014-2015) in local markets of Aizawl city, and the price is increasing day by day.

Feeding alone accounts for 60-80% of the total production cost of broiler enterprise. This factor has been the main hindrance for broiler farmers in Mizoram toward achieving the expected monetary returns from their enterprises. The cost of both broiler commercial feed mixture and feed ingredients is high in Mizoram as compared to other states of the mainland. It is because of geographical constraints resulting in deficit production of food grains, and hilly terrains causing higher transportation cost and hence the prices of the commodities. To ensure remunerative return to broiler farmers in Mizoram, it has become imperative to search for alternative feed resources which do not have competition with human being for consumption to reduce the feeding cost of broilers.

Utilization of unconventional local feeds as substitutes of conventional ones is a popular trend in broiler feeding practices [1-4]. Depending on availability and nutritional values, many unconventional feeds are used in broiler rations to economize the feeding. In Mizoram, *Tithonia diversifolia*, i.e., wild sunflower may be one such promising unconventional feed as it is abundantly available throughout Mizoram and is a good source of protein and rich in other nutrients [5]. Wild sunflower or Mexican sunflower, also known as tree marigold, belongs to family “Asteraceae” and botanical name is *T. diversifolia* (Hemsl.) A. Gray. In Mizoram, it is locally called as *Bawng-pu-pang-par* or *Vai-va-kawn-par*. It is a large shrub with 3-5 lobed leaves and yellowish orange flowers. It grows well at altitude 800-1500 m and flowering season is November and December month of every year. The flower heads are used in wounds and bruises. The plant is also used as remedy against malaria, jaundice, hepatitis, liver problems, intestinal parasites, sore throat, etc. The leaves are sometimes collected for cattle fodder and also cooked with other leaves for pig’s feed [6]. The leaves of *T. diversifolia* are good sources of nutrients, particularly protein, energy, and mineral matters. It has 22.47±0.34%dry matter (DM), and 25.07±0.00% crude protein (CP), 2.19±0.00% ether extract (EE), 9.51±0.01% total ash (TA), and 4223±4.04 kcal GE/kg on DM basis [7]. It contains 1.72±0.02% calcium, 0.17±0.00% total phosphorous, 0.12±0.00 ppm copper, 0.87±0.03 ppm iron, and 1.66±0.05 ppm manganese. The acid detergent lignin, total tannins, and condensed tannins of TDLM are 7.93±0.39%, 5.79±0.26%, and 0.23±0.01%, respectively [8].

Considerable information is available about utilization of TDLM for broiler feeding in other parts of the world [9,10], but no report is available about its utilization as broiler feed substitute in Mizoram and rest of Indian subcontinent. The findings generated in other parts of the world may not be directly applicable to the farmers of Mizoram, and India as a whole, for differences in altitudes and agro-climatic conditions. Therefore, in this study, an attempt was made to evaluate the effects of feeding *T. diversifolia* leaf meal (TDLM) on growth performance, nutrient utilization and feed efficiency of broilers reared under deep litter system of management in Mizoram.

## Materials and Methods

### Ethical approval

The necessary approval was taken from Institutional Animal Ethics Committee (Approval reference number-CVSC/CAU/IAEC/11-12/P2) for conducting the study.

### Location of the study

The study was carried out at the poultry unit of Instructional Livestock Farm Complex (ILFC), College of Veterinary Sciences and Animal Husbandry, Central Agricultural University, Selesih, Aizawl, Mizoram, India.

### Collection of T. diversifolia leaves and preparation of leaf meal

**Figure-1:**
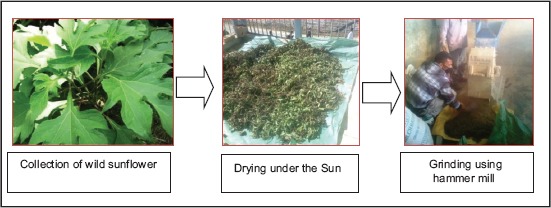
Steps involved in preparation of *Tithonia diversifolia* leaf meal.

The wild sunflower (*T. diversifolia*) plants were collected from different locations in and around the College of Veterinary Sciences and Animal Husbandry, Central Agricultural University, Aizawl, Mizoram. The leaves of different developmental stages were separated from stems, pooled together and sun-dried until the leaves become crispy while still retaining the greenish coloration. The dried leaves were then milled using a hammer grinding machine to produce leaf meal and stored in airtight condition until utilization (Figure-1).

### Experimental birds and design of experiment

A total of 180, 1-day-old broiler chicks were purchased from retailer of Aizawl city, Mizoram, India. The chicks were randomly divided into six homogeneous groups with 30 chicks in each group in a completely randomized design. Each group had three replicates of 10 chicks in each. The experimental groups were designated as TDLM-0, TDLM-2, TDLM-4, TDLM-6, TDLM-8, and TDLM-10. The birds were wing-banded for easy identification.

### Ration formulation and feeding management

Isocaloric and isonitrogenous broiler starter and finisher rations were formulated (Table-1) following the recommendations of BIS [11]. Starter ration was fed up to 35^th^ day of age and finisher ration, thereafter, was up to 42^nd^ day of age. The TDLM was incorporated in starter and finisher rations at 0% (TDLM-0), 2% (TDLM-2), 4% (TDLM-4), 6% (TDLM-6), 8% (TDLM-8), and 10% (TDLM-10) level of the total ration. The quantities of conventional ingredients (yellow maize, rice polish, soybean meal, and groundnut cake) were adjusted to balance the rations.

**Table-1:** Ingredient (kg) and nutritional composition (% on DM basis) of the experimental rations with TDLM.

Ingredient	TDLM-0		TDLM-2		TDLM-4		TDLM-6		TDLM-8		TDLM-10	
					
	BS	BF	BS	BF	BS	BF	BS	BF	BS	BF	BS	BF
Ingredient composition												
YM	52	57	52	57	52	56	51	56	50	56	50	55
RP	10	13	9	12	9	12	9	11	9	10	8	10
SBM (SE)	20	15	20	14	18	13	17	13	16	12	16	12
GNC (SE)	10	8	9	8	9	8	9	7	9	7	8	6
FM	6	5	6	5	6	5	6	5	6	5	6	5
TDLM	0	0	2	2	4	4	6	6	8	8	10	10
FA/MM*	1.8	1.8	1.8	1.8	1.8	1.8	1.8	1.8	1.8	1.8	1.8	1.8
Salt	0.2	0.2	0.2	0.2	0.2	0.2	0.2	0.2	0.2	0.2	0.2	0.2
Total	100	100	100	100	100	100	100	100	100	100	100	100
Nutritional composition (% on DM basis)												
DM	91.65	91.18	91.17	91.2	91.26	88.79	91.18	90.84	91.35	91.08	91.38	91.13
CP	23.58	20.68	23.41	20.49	23.3	20.52	23.26	20.35	23.2	20.26	23.05	20.2
EE	3.88	4.37	3.77	4.3	3.8	4.32	3.81	4.24	3.81	4.17	3.71	4.19
CF	4.2	4.2	4.32	4.33	4.48	4.62	4.69	4.78	5.15	5.02	5.2	5.2
TA	13.06	10.05	12.55	11.14	12.34	10.74	11.94	10.17	12.99	11.46	11.32	11.7
NFE	55.28	60.7	55.95	59.74	56.08	59.8	56.3	60.46	54.85	59.09	56.72	58.71
ME	2879	2939	2874.1	2931.6	2870.4	2921.2	2860	2916.3	2849.9	2908.9	2845	2901
Ca	0.96	0.87	0.99	0.89	1.02	0.93	1.05	0.95	1.09	0.99	1.11	1.01
AP	0.48	0.45	0.48	0.44	0.47	0.44	0.47	0.44	0.46	0.42	0.46	0.41
L	1.39	1.2	1.37	1.16	1.29	1.12	1.25	1.07	1.21	1.03	1.2	1.02
M	0.64	0.6	0.63	0.59	0.62	0.58	0.61	0.57	0.6	0.56	0.59	0.56

BS=Broiler starter, BF=Broiler finisher, YM=Yellow maize, RP=Rice polish, SBM (SE)=Soyabean meal (solvent extracted), GNC (SE)=Ground nut cake (solvent extracted), FM=Fish meal, FA/MM=Feed additives/mineral mixture. *Feed additives/mineral mixture: TCP=1.0%, trace mineral mixture=0.4%, Vitamin mixture=0.2%, Lysine=0.05%, Methionine=0.15%. DM=Dry matter, CP=Crude protein, EE=Ether extract, CF=Crude fiber, TA=Total ash, NFE=Nitrogen free extract, ME=Calculated value in kcal/kg, Ca=Calcium, AP=Available phosphorous, L=Lysine, M=Methionine, Quantity of Micro-mineral added: 0.4 kg/100 kg feed mixture, Quantity of vitamins added=0.2 kg/100 kg feed mixture, TCP=Tricalcium phosphate, TDLM=*Tithonia diversifolia* leaf meal

The feed was provided *ad libitum* to the experimental birds and drinking water was made available throughout the day. The chicks were reared in battery brooder during the first 2 weeks of their life and afterward housed in well-ventilated deep litter house partitioning into 18 hens providing required floor space (1.5 ft×1.5 ft/bird). Rice husk was utilized as litter material. The birds were vaccinated against new castle disease (Lasota) on the 7^th^ day and against infectious bursal disease on the 14^th^ day of age. The strict biosecurity measures were followed during the experiment.

### Duration of experiment and details of records made

The feeding trial was conducted for 6 weeks. The total amounts of feed offered and residue left within 24 h were recorded daily for calculation of average daily feed intake. The individual body weights were taken at weekly interval. Mortality record was made, and post-mortem examination was carried out to find out the probable cause of death.

### Metabolic trial

At the end of 6^th^ week, a metabolic trial for 3 days was conducted. Two birds from each replicate, i.e., six birds from each treatment were randomly selected for the trial. The feed offered and residue left within 24 h was recorded to estimate daily feed intake. Feces voided within 24 h were collected and weighed. A definite proportion of feces (i.e. 1/20^th^ part) after thorough mixing was kept in glass petridish and placed in hot air oven at 100±1°C overnight for determination of DM. Another sample, 1/30^th^ part of feces, was weighed and preserved in 1:4 H_2_SO_4_ for determination of nitrogen. After 3 days of collection, the pooled samples of feces were mixed thoroughly and a suitable amount of undried preserved samples was weighed in duplicate for determination of nitrogen. The dried sample after grinding was utilized for estimation of other proximate principles.

### Chemical assay

The rations, residual feeds, and feces voided during the metabolic trial were analyzed for DM, crude fiber (CF), EE, nitrogen free extract (NFE) and TA following methods of AOAC [12] and nitrogen for CP estimation by Kjeldahl method. The calcium and phosphorous were estimated as per methods of Talapatra *et al*. [13]. The micro minerals were analyzed in atomic absorption spectrophotometer (GBC) following standard protocols. The tannin was estimated as tannic acid equivalent using Folin–Ciocalteu reagent method [14] and condensed tannin as leucocyanidin equivalents following method of Porter *et al*. [15].

### Statistical analysis

The generated data were subjected to analysis of variance following statistical methods of Snedecor and Cochran [16].

## Results and Discussion

### Feed consumption

Feed is the most costly expense in broiler production involving 60-80% of the total production cost. Thus, the feed efficiency is typically the primary tool by which performance of a broiler flock is evaluated. The average daily feed intake of broilers fed TDLM is presented in Table-2. Daily feed intake decreased with increased inclusion level of TDLM in the ration. However, no significant difference (p>0.05) was observed among the groups in average daily feed intake except during the 3^rd^ week of age. Decreasing trend of feed consumption might be for increasing CF levels with increased level of TDLM in the ration resulting bulkiness thereby reducing feed consumption. Similar findings were also reported by Ekeocha [9] for broiler. Odunsi *et al*. [17] reported that broiler finishers fed wild sunflower leaf meal showed depressed feed intake most especially at 7.5 and 10.0% inclusion level. Decreased feed intake might also be for anti-nutritional factors present in TDLM, particularly tannin that consequently made rations bitter as it increased across treatments [9].

**Table-2:** Average daily feed intake (g/bird/day) in broilers fed TDLM.

Age	TDLM-0	TDLM-2	TDLM-4	TDLM-6	TDLM-8	TDLM-10
1^st^	18.03±2.00	16.99±1.86	16.41±2.36	15.80±2.20	15.44±2.16	14.74±1.65
2^nd^	42.37±4.74	41.07±4.13	40.49±4.34	40.70±4.59	36.26±3.38	34.88±4.10
3^rd^	63.12±2.96^a^	61.16±3.05^abc^	60.72±2.51^abc^	62.41±2.38^ab^	54.97±1.32^bc^	53.37±2.43^c^
4^th^	88.23±7.31	86.50±6.51	86.56±5.87	86.27±6.25	76.15±4.95	74.68±3.37
5^th^	137.92±5.94	136.88±5.46	135.10±2.02	134.63±3.45	129.09±4.42	127.72±5.64
6^th^	149.89±8.17	149.43±4.80	151.25±4.02	146.75±5.19	144.30±3.76	143.73±2.63

Means bearing different superscript (a, b, c) in a row differ significantly (p<0.05). TDLM=*Tithonia diversifolia* leaf meal

### Growth performance of broilers

The average daily gain in body weight decreased with increased inclusion level of TDLM in the ration (Table-3). However, the growth rate was comparable up to 4% inclusion level without any significant difference, but reduced significantly (p<0.05) at and above 6% level of TDLM. Togun *et al*. [18] reported that live and carcass weight of broilers were reduced significantly by wild sunflower forage meal inclusion above 10% level in the ration. Similarly, Bolu *et al*. [4] also observed reduced growth rate in broilers when fed dried pawpaw seed above 5% level in the ration. The poor growth performance of the birds with higher inclusion levels of TDLM might be for accumulative or chronic effects of the anti-nutritional factors present in TDLM.

**Table-3:** ADG in body weight (g/bird) in broiler fed TDLM.

Age	TDLM-0	TDLM-2	TDLM-4	TDLM-6	TDLM-8	TDLM-10
1^st^	9.96^a^±0.21	9.45^a^±0.24	8.67^b^±0.23	7.99^c^±0.28	6.18^d^±0.20	5.12^e^±0.25
2^nd^	35.74^a^±1.07	35.60^a^±1.13	33.92^a^±1.06	26.43^b^±0.88	19.57^c^±1.05	15.68^d^±0.72
3^rd^	54.43^a^±1.24	52.24^ab^±0.90	50.94^b^±0.92	30.74^c^±0.98	28.85^c^±0.44	20.86^d^±0.91
4^th^	37.00^a^±0.87	36.22^ab^±0.89	36.21^ab^±1.74	32.86^c^±1.19	31.91^b^±2.64	26.63^d^±1.06
5^th^	39.12^a^±0.92	39.01^a^±0.94	40.56^a^±1.05	31.85^b^±0.90	35.31^c^±1.30	32.07^b^±1.37
6^th^	46.67^ab^±1.63	46.26^ab^±1.22	48.54^a^±0.96	44.29^bc^±0.54	41.30^c^±0.92	43.71^bc^±1.33

Means bearing different superscripts (a, b, c, d, e) in a row differ significantly (p<0.05). ADG=Average daily gain, TDLM=*Tithonia diversifolia* leaf meal

The average body weight at 42^nd^ day of age was recorded as 1624.72±30.52, 1596.66±17.84, 1592.60±19.24, 1404.61±17.76, 1188.29±17.67, 1054.33±18.81 g/bird, respectively, for TDLM-0, TDLM-2, TDLM-4, TDLM-6, TDLM-8, and TDLM-10 (Table-4). The average body weights of TDLM-0 and TDLM-2were significantly higher (p<0.05) than other groups up to 21^st^ day of age, but from 28^th^ day onward, no significant difference (p>0.05) was observed among between TDLM-0, TDLM-2, and TDLM-4 and was significantly (p<0.05) higher than TDLM-6, TDLM-8, and TDLM-10. This might be for depressed feed acceptability and consequently nutrient intake [19] for high fiber and bulkiness of feed mixture in TDLM-6, TDLM-8, and TDLM-10. Similar findings were also reported by Akinmutimi *et al*. [20] for sword bean (*Canavalia gladiata*) and Obun *et al*. [21] for *Detarium microcarpum* seed meal in broilers.

**Table-4:** Average weekly body weight (g/bird) of broiler fed TDLM incorporated ration.

Age	TDLM-0	TDLM-2	TDLM-4	TDLM-6	TDLM-8	TDLM-10
0 day	47.81±0.32^NS^	47.79±0.57^NS^	47.62±0.36^NS^	47.73±0.33^NS^	47.53±0.35^NS^	47.73±0.28^NS^
7^th^ day	117.51±1.77^a^	113.91±2.22^a^	108.29±1.94^b^	103.78±2.26^b^	91.26±1.77^c^	83.76±2.00^d^
14^th^ day	367.68±9.11^a^	363.14±10.04^a^	345.74±9.32^a^	291.01±7.82^b^	227.08±8.78^c^	192.43±6.61^d^
21^st^ day	753.90±15.26^a^	728.85±15.90^ab^	707.55±12.79^b^	504.40±12.94^c^	429.84±9.28^d^	338.49±12.26^e^
28^th^ day	1012.94±17.05^a^	982.38±19.81^a^	968.63±22.10^a^	867.44±20.14^b^	643.55±24.52^c^	524.90±18.63^d^
35^th^ day	1298.03±21.01^a^	1263.19±13.37^a^	1252.83±16.45^a^	1090.69±15.75^b^	899.16±19.74^c^	748.33±26.84^d^
42^nd^ day	1624.72±30.52^a^	1616.66±17.84^a^	1592.60±19.24^a^	1404.61±17.76^b^	1188.29±17.67^c^	1054.33±18.81^d^

Means bearing different superscripts (a, b, c, d, e) in a row differ significantly (p<0.05). TDLM=*Tithonia diversifolia* leaf meal

### Digestibility of nutrients

Apparent digestibility of nutrients (Table-5) decreased for the inclusion of TDLM in the ration. The digestibility coefficient of CP, EE, CF, and NFE ranged from 53.51±2.12% to 65.89±0.05%, 49.46±1.21% to 61.40±0.49%, 42.98±2.71% to 43.92±3.40%, and 69.36±2.41% to 82.09±0.51%, respectively. The decreasing trend of digestibility in TDLM included groups might be for high CF contents and anti-nutritional factors of TDLM. Tannin can bind dietary proteins and digestive enzymes into complexes which are not readily digestible. It can also bind with the proteins of saliva and mucosal membranes [22]. However, no significant differences (p>0.05) were observed among groups in CF digestibility but was in decreasing trend.

**Table-5:** Nutrient digestibility (%) in broiler fed TDLM incorporated rations.

Nutrient	TDLM-0	TDLM-2	TDLM-4	TDLM-6	TDLM-8	TDLM-10
DM	73.88±0.83^a^	70.47±0.67^ab^	67.99±1.06^abc^	64.68±3.31^bc^	62.08±3.02^c^	61.64±3.11^c^
CP	65.89±0.05^a^	63.53±1.04^ab^	62.28±1.62^ab^	59.43±0.21^b^	53.51±2.12^c^	53.53±2.33^c^
EE	61.40±0.49^a^	54.63±4.23^abc^	58.68±1.13^ab^	60.83±0.56^a^	52.34±2.36^bc^	49.46±1.21^c^
CF	44.16±1.81	43.92±3.40	42.98±2.71	43.86±1.52	43.65±2.23	43.11±2.67
NFE	82.09±0.51^a^	76.84±0.13^ab^	73.75±0.35^b^	70.13±4.60^b^	69.78±2.19^b^	69.36±2.41^b^

Means bearing different superscripts in a row differ significantly (p<0.05). TDLM=*Tithonia diversifolia* leaf meal, DM=Dry matter, CP=Crude protein, EE=Ether extract, CF=Crude fiber, NFE=Nitrogen free extract

### Mortality

No mortality was recorded up to 8% TDLM inclusion level, but one bird died (3.13% mortality) in TDLM-10. However, on post-mortem examination, no significant lesions were noticed in spleen, liver, and kidneys. This might be indication that death might not be for toxic effects of TDLM.

### Feed conversion ratio (FCR)

The mean FCR at 42^nd^ day of age was estimated as 2.17±0.15, 2.17±0.15, 2.13±0.13, 2.46±0.16, 2.66±0.11, and 3.96±0.10, respectively, for TDLM-0, TDLM-2, TDLM-4, TDLM-6, TDLM-8, and TDLM-10. During the 1^st^ to 6^th^ week, the FCR decreased with increased inclusion level of TDLM. However, there was no significant difference up to 4% TDLM inclusion level. Decreasing trend in FCR might be for reduced body weight gain due to decreased digestibility of nutrients for inclusion of TDLM. Prince *et al*. [23] also observed depression in growth rates of chicks when fed diet containing high tannin sorghum.

## Conclusion

It was concluded from the findings of this study that TDLM could be incorporated up to 4% level without any adverse effects on growth performance, nutrient utilization, and feed conversion efficiency of broilers reared under deep litter system of management in Mizoram.

## Authors’ Contributions

The study was designed and carried out by RB as a part of an Intramural Research project financed by Central Agricultural University, Imphal, Manipur, India. RB drafted, revised and approved the final manuscript.
